# The effect of radiotherapy on taste sensation in head and neck cancer patients – a prospective study

**DOI:** 10.1186/s13014-020-01578-4

**Published:** 2020-06-05

**Authors:** Michal Asif, Assaf Moore, Noam Yarom, Aron Popovtzer

**Affiliations:** 1 Sackler Faculty of Medicine, Tel Aviv University, Tel Aviv, Israel; 2 Institute of Oncology, Davidoff Center, Rabin Medical Center – Beilinson Hospital, Petach Tikva, Israel; 3 Oral Medicine Unit, Sheba Medical Center, Tel Hashomer, Israel; 4 School of Dental Medicine, Tel Aviv University, Tel Aviv, Israel

**Keywords:** Head and neck cancer, Radiotherapy, Taste sensation, Sialometry, Xerostomia

## Abstract

**Background:**

One of the main side effects of head and neck (H&N) radiation therapy (RT) is alteration in taste sensation. It causes significant morbidity and has a major effect on quality of life (QoL). The aim of this study was to prospectively define the effect of RT on taste sensation (general, and four basic tastes) and correlate these findings with changes in saliva secretion and QoL questionnaires.

**Methods:**

Patients with H&N cancer treated with RT, in which the oral cavity was expected to receive a mean dose of 30 Gray (Gy). Patients were evaluated by Whole-Saliva Sialometry, validated Taste Strips and European Organization for Research and Treatment of Cancer H&N QoL questionnaires prior to RT (T0), mid-point of radiotherapy dose (T1), at the end of radiotherapy (T2) and 1 (T3), 3 (T4) and 12 months (T5) after completion of treatment course.

**Results:**

Twenty-eight patients were recruited, and 21 patients completed study procedures and were analyzed. Median age was 66 years (range 18–90). The most common tumor site was the oral cavity. The median prescribed radiation dose to the high dose volume was 66 (range 60–70). The median mean and max dose to the oral cavity were 25.1 (range 14–69) and 64.9 (range 30–70), respectively. There was a significant decrease in overall taste sensation between T0 and T1 and T2. With specific tastes, there were significant decreases in sensation of sweet and salty, a trend with bitter and no change with sour. All returned to baseline at T3 and onwards. There was no significant correlation between the max or mean dose to the oral cavity and overall taste sensation or between doses to different areas of the tongue and overall or specific tastes. At T0 there was a significant positive correlation between overall taste sensation and whole-saliva sialometry, and at T1 and T2 there were strong trends. There were significant declines in QoL scores during RT.

**Conclusions:**

We found a significant immediate reduction in taste sensation due to RT in H&N cancer patients with taste recovery 1 month after treatment completion. There were strong trends to a correlation with saliva production that requires further exploration.

## Background

The standard treatments for most Head and Neck (H&N) cancers include surgery, radiotherapy (RT), and chemotherapy (CT). One of the main adverse events of these treatments is altered taste sensation [1–16] which has a major effect on quality of life (QoL). It is a significant cause of morbidity and affects patients’ diet by causing loss of appetite, reduced oral intake, and may result in weight loss [8, 17, 18].

The sense of taste is mediated by taste buds, found primarily on the dorsum of the tongue, but also on the lips, cheeks, palate, oropharynx and larynx. The sensation of taste includes five established basic tastes, including bitter, sweet, sour, salty and umami. Taste buds can be found on the tongue, soft palate, lip and buccal mucosa, pharynx, larynx, uvula, and upper third of the esophagus [8]. Taste buds on the tongue appear as specialized structures known as circumvallate, foliate, and fungiform papillae; each containing complexes of 50–100 taste receptors [8]. Taste buds may be short- and long-lived cells. Some populations were estimated to have half-lives of 8–12 days, others have half-lives in the region of 24 days [19, 20]. Taste changes include hypogeusia (reduced taste sensation), dysgeusia (altered taste sensation), and ageusia (loss of taste sensation) [3, 4]. Basic tastes can be perceived in all areas of the tongue where taste buds are located [1, 6].

Previous studies found significant taste loss 4–5 weeks after starting RT treatments [1–3, 7], but the recovery rate is still controversial. Some studies reported that most patients recover 1–4 months after RT [15, 17, 21], while others showed incomplete or no recovery even several years later [6–8]. Furthermore, few studies have been conducted to estimate the effect of glossectomy on taste sensation in tongue cancer patients [6, 8]. Taste disorders were found to be significantly associated with glossectomy. The recovery rate and improvement in QoL are dependent on the extent of the resection and remaining volume of the tongue [21].

Alterations in saliva flow rate have been suggested by some reports to be one of the main causes for taste change [22], while others have had contradictory results [3–6]. Saliva contributes to oral health and basic functions such as moistening the oral cavity, mastication, swallowing, food digestion, facilitating speech, helping protecting oral mucosa, and remineralizing hard dental tissues. Saliva is produced by major and minor glands that are highly susceptible to radiation, which causes destruction of glandular cells and hypofunction. Salivary glands hypofunction and xerostomia are known iatrogenic side effects of head and neck RT [13, 23]. However, xerostomia’s correlation with taste loss is still unclear [2–4, 6].

Most previous studies of taste changes in oncology were limited to specific modalities, i.e., RT and CT, with few studies including patients who underwent surgery as treatment for tongue cancer. Moreover, mainly short-term follow-ups of taste loss recovery were documented (generally a few weeks). Finally, previous studies used non-validated techniques usually prepared by the research group itself or a related laboratory [24, 25].

The aim of this study was to identify the effect of RT on taste sensation (general taste sensation and four basic tastes - bitter, sweet, sour and salty) and correlate these findings with changes in saliva secretion and QoL. Some studies reported on a discrepancy between patients’ taste perceptions and actual measured taste loss [6], potentially due to adaptation to the sensory loss. We aimed to compare differences between baseline, during and post RT, by repeated measurements using validated techniques.

### Study objectives and hypothesis

In this study, we aimed to evaluate the recovery rate of taste sensation at 1, 3 and 12 months follow-up after end of treatment, to examine the correlation between changes in taste sensation and differences in saliva volumes, to investigate the associations between radiation dosage to oral cavity and taste, to study the correlations between radiation dose to the taste buds and taste alteration, and to study the correlation between QoL and changes in taste sensation.

We hypothesized that a significant difference in taste test scores would be found at the end of treatment compared to baseline and at 1- and 3-months follow-ups post treatment. We also hypothesized that a significant correlation would be found between saliva volume and changes in taste sensation, and that a significant correlation would be revealed between QoL score and changes in taste sensation. We believed the mean dose to the oral cavity could be associated with a chronic effect on taste.

## Methods

### Patients

Included were 28 patients, 18 years or older, with recently diagnosed H&N cancer who were scheduled for adjuvant or definitive treatment at the H&N Unit, Davidoff Cancer Center, Rabin Medical Center, who had signed informed consent. Excluded were patients who had undergone total glossectomy, had a prior diagnosis of diseases effecting saliva secretion or causing salivary glands impairment (i.e., Sjogren’s syndrome, iodine cancer treatment), had a reported history of abnormal sense of taste or eating disorders, were current heavy smokers (smoke more than one pack/day) or previous heavy smokers (stopped smoking during the last 2 years and had smoked more than one pack/day).

### Data collection

The individual medical records were reviewed for patients’ clinicopathological characteristics, treatment details, and past medical history.

### Study procedures

Patients were evaluated at baseline prior to RT (T0), mid-point of RT course (T1), end of RT (T2), and approximately 1-, 3- and 12-months post RT (T3, T4 and T5, respectively). At each encounter, patients underwent an oral evaluation that included soft tissue examination, objective tests including Whole-Saliva Sialometry, and validated Filter Paper Strips Taste Test (“Taste Strips”, Burghart, Wedel, Germany), and responded to European Organisation for Research and Treatment of Cancer (EORTC) QoL questionnaires (C30 and H&N43).

Whole-saliva sialometry test, measures the subject’s unstimulated and stimulated whole saliva volume in order to provide a quantitative objective assessment of salivary glands (major and minor) function. First, a subject’s unstimulated whole saliva was collected into specific pre-weighed test tubes (using the same analytical scale) for 5 min. The subject was then asked to collect saliva and spit during the test in a one-minute intervals. Stimulation of saliva was performed using 2% citric acid that was applied on the lateral aspects of tongue using swabs while measuring 30 s intervals for 2 min (seconds 0, 30, 60, 90, 120). One minute after applying the 2% citric acid, the subject’s saliva was collected over 5 min in the same fashion previously described. The quantity of saliva was measured by weighing test tubes again 1 hour after the test at the most [26].

After sialometry, patients rinsed with distilled water. Then, the validated filter paper strips taste test was conducted. The kit includes 18 different taste strips, presented to the subject successively in a pseudo-randomized manner (taste and concentration are known to the examiner only), which evaluates the four basic tastes (bitter, sweet, sour and salty) in four increasing concentrations in a manner of a forced choice testing [27]. This test is incapable of assessing umami. Included are strips impregnated with the four tastes, as well as control strips that contain no taste. The strip length is 8 cm, the tip area is 2 cm^2^ and is impregnated with a tastant (4 concentrations each of the 4 basic taste qualities). The following concentrations and tastants were used for the taste strips: sweet: 0.4, 0.2, 0.1, 0.05 g/ml sucrose; sour: 0.3, 0.165, 0.09, 0.05 g/ml citric acid; salty: 0.25, 0.1, 0.04, 0.016 g/ml sodium chloride; bitter: 0.006, 0.0024, 0.0009, 0.0004 g/ml quinine hydrochloride. The strips were introduced to the central tongue in increasing concentrations. The order for each cycle of strip administration was; control, sweet, sour, salty and bitter. Each correct answer yields one point. If all 16 strips are identified correctly the maximum score is 16 points (4 points for each basic taste. Control strips are not counted during evaluation). The general taste score consists of 0–16 points and was evaluated for each patient in a specific time. The specific taste score consists of 0–4 points. Threshold alteration in concentrations were compared to baseline concentrations as determined in the first encounter (T0).

Toxicity classification and assessment were done in accordance with the Common Terminology Criteria for Adverse Events (CTCAE) v4.3 grading [28]. These included: dysgeusia grading, with an adverse event scale of 1–2; dry mouth (xerostomia) grading with an adverse event scale of 1–3 and oral mucositis grading (with 1–5 scale) [28].

Patients completed the EORTC quality of life questionnaires (C30 and H&N43) for QoL assessment [29]. The QoL questionnaire consists of two parts: The QLQ-C30 with 30 questions (Q1-Q30) and HN43 questionnaire with 43 additional questions relevant for H&N cancer patients (Q31-Q73). All questions have a similar structure of 1–4 scoring system (1 = Not at all, 4 = Very much), excluding questions 29 (“How would you rank your general health status in the last week?”) and 30 (“How would you rank your general quality of life in the last week?”) which have a 1–7 scoring system of opposite direction (1 = Very bad, 7 = Excellent) and thus, needed to be statistically evaluated separately. Both questionnaires were translated to Hebrew and validated by the EORTC quality of life group.

### RT planning and dose-volume histogram (DVH) evaluation

Anatomical areas of interest were contoured using the Eclipse® treatment planning system (Varian Medical Systems, Palo Alto, CA, USA). Dose calculations were determined using the AAA algorithm version 8. Patients were treated with intensity-modulated radiation therapy (IMRT)-based RT using dynamic sliding window multileaf collimator (MLC) or volumetric modulated arc therapy (VMAT). Quality assurance verification plans were performed with the ArcCHECK® dosimeter (Sun Nuclear Corporation, Melbourne, Florida, USA). Doses to anatomical areas of interest were collected from the DVHs of approved treatment plans.

### Statistical analysis

A sample size of 25 subjects was needed in order to detect a mean difference of 1.5 units between two taste kit scores (total taste score) to obtain a power of 0.96 (*P* < 0.05, 2-tailed). The determination of the required sample size was made by the Laboratory of Statistical Consultation, School of Mathematical Sciences, Tel-Aviv University. Data were analyzed with IBM SPSS Statistics version 23. A *P*-value of 0.05 or less was considered statistically significant. Correlations and regression analysis were performed to assess relationships between changes in taste sensation (dependent variable) and differences in saliva volumes, with QoL questionnaire and dose volume histogram (independent variables).

The research protocol was approved by the Institutional Review Board (0295–15-RMC) prior to any research procedures.

The greater part of this study was conducted as part of the M. Sc program at the School of Graduate Studies, Sackler Faculty of Medicine, Tel Aviv University.

## Results

### Patient, tumor and treatment characteristics

During 2016–2018, 28 patients were recruited of whom 21 completed the required study procedures and were included in the study. The median age was 66 (range 18–90) years. Median patient weight was 74 and 73 kg, prior to and 1 month after RT, respectively. The median weight loss during treatment was 1.5 (range 0–6.4) kilograms. The most common tumor site was the oral cavity in 7(28%) of patients, followed by major salivary gland tumors (6, 24%), H&N skin tumors (mostly parotid squamous cell carcinoma) (5, 20%) and oropharynx (1, 4%), thyroid (1,4%) and esthesioneuroblastoma (1, 4%). In 88% of cases, RT was delivered in the adjuvant setting, the remaining patients were treated with definitive intent. The median prescribed dose for the high dose volume was 66 (range 60–70) Gy. All patients were treated with daily 2 Gy fractions. The total duration of treatment was 6–7 weeks. The median mean and max dose to the oral cavity were 25.1 (range 14–69) and 64.9 (range 30–70) Gy, respectively (Table 1).

**Table 1:** Patient and treatment characteristics

Patient number	Smoking history	Ethylism	Primary tumor site	Surgery of oral cavity	Radiotherapy technique and total dose	Concurrent chemotherapy
1	No	No	Oropharynx	Yes	VMAT, 66Gy	No
2	Yes	No	Salivary Glands	Yes	VMAT, 60Gy	No
3	No	No	Oral Cavity	Yes	IMRT, 66Gy	No
4	Yes	No	Oral Cavity	Yes	VMAT, 60Gy	No
5	No	No	Salivary Glands	Yes	IMRT, 66Gy	No
6	No	No	Oropharynx	No	VMAT, 70Gy	No
7	No	No	Salivary Glands	Yes	IMRT, 66Gy	No
8	No	No	Thyroid	Yes	IMRT, 66Gy	No
9	No	No	Nasal Cavity	Yes	IMRT, 60Gy	No
10	Yes	No	Salivary Glands	Yes	VMAT, 66Gy	No
11	Yes	No	Salivary Glands	Yes	IMRT, 60Gy	No
12	No	No	Oral Cavity	Yes	IMRT, 66Gy	No
13	Yes	No	Skin	Yes	VMAT, 60Gy	No
14	Yes	Yes	Salivary Glands	Yes	IMRT, 70Gy	No
15	No	No	Oral Cavity	Yes	IMRT, 60Gy	No
16	No	No	Skin	No	IMRT, 70Gy	No
17	No	No	Salivary Glands	Yes	VMAT, 66Gy	No
18	Yes	No	Oral Cavity	Yes	VMAT, 60Gy	No
19	No	No	Skin	No	IMRT, 70Gy	No
20	No	No	Oral Cavity	Yes	IMRT, 66Gy	No
21	Yes	No	Oral Cavity	Yes	VMAT, 60Gy	No

*VMAT* Volumetric modulated arc therapy, *IMRT* Intensity-modulated radiation therapy

### Taste sensation

There was a significant decrease of 2 points in overall taste sensation between T0 and T1 (*P* = 0.050), and of 3.28 points between T0 and T2 (*P* = 0.01).

**Fig. 1:**
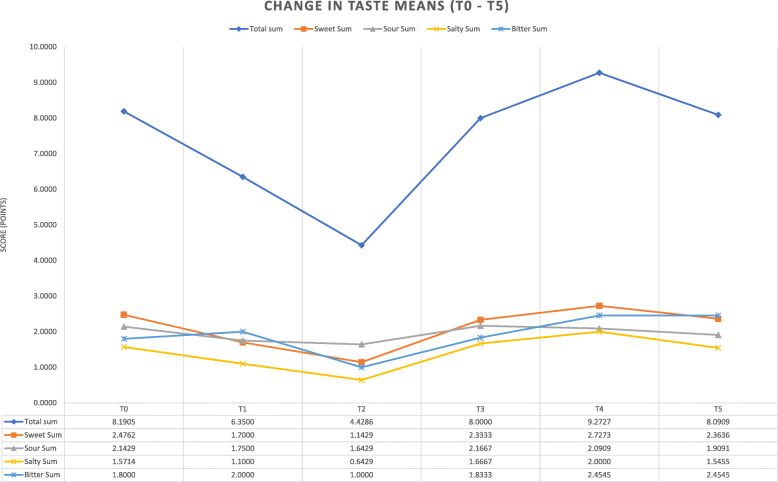
Trends in Taste Sensation Over One Year. The X axis is the evaluation point in time: prior to radiotherapy (T0), mid-point of radiotherapy course (T1), at the end of radiotherapy (T2), and approximately 1,3, and 12 months post radiotherapy (T3, T4 and T5 respectively). Each graph represents a specific taste’s median value (sweet sum = sweet, sour sum = sour, salty sum = salty, bitter sum = bitter) of overall taste sensation (total sum). The Y axis represents the median score in the validated Filter Paper Strips Taste Test. A decline can be clearly seen at the end of the radiotherapy course (T2) and a rapid recovery of taste sensation at T3–4

There was a significant decrease of 1.14 points in sweet taste sensation between T0 and T2 (*p* = 0.020) and a trend of 0.85 points between T0 and T1 (*p* = 0.053). There was a significant decrease of 1.14 points in salty taste sensation between T0 and T2 (*p* = 0.040). There was a trend for decrease of 0.85 points in bitter taste sensation between T0 and T2 (*p* = 0.068). There was no significant change with sour taste sensation. There were no significant differences in taste sensation between T3, T4 and T5 compared with T0 in overall taste sensation or specific tastes (Fig. 1).

### Correlation between tongue dose and taste sensation

We did not find any significant correlation between the maximum or mean dose to the oral cavity and overall taste sensation. There was also no significant correlation between doses to different areas of the tongue, and overall or specific tastes (Table 2).

**Table 2:** Probability values (*p*-values) for Correlations Between Alteration in Taste Sensation and Examined Factors

Factor	T0	T1	T2	T3	T4	T5
Non-stimulated sialometry	0.033	0.17	0.058	0.386	0.349	0.782
Alteration in citric-acid-stimulated sialometry	0.007	0.055	0.237	0.255	0.12	0.747
Maximal tongue dose	0.884	0.157	0.429	0.748	0.226	0.416
Mean tongue dose	0.913	0.183	0.394	0.475	0.303	0.636
Maximal oral cavity dose	0.981	0.816	0.902	0.540	0.907	0.722
Mean oral cavity dose	0.854	0.237	0.407	0.451	0.798	0.617

T0, baseline (prior to radiotherapy course); T1, mid-point of radiotherapy course; T2, end of radiotherapy; T3, 1 month after completion of treatment course; T4, 3 months after completion of treatment course; T5, 12 months after completion of treatment course

### Correlation between mucositis and taste sensation

We did not find any significant correlations between the degree of mucositis and the overall and specific taste sensation during the course of RT. There was a correlation between the grade of mucositis and overall taste sensation only at T4 (*P* = 0.049).

### Whole-saliva sialometry

At T0, there was a significant positive correlation between overall taste sensation and Whole-Saliva Sialometry with and without citric acid stimulation (*P* = 0.007 and *P* = 0.033, respectively).

There were trends for a positive correlation between overall taste sensation and stimulated Whole-Saliva Sialometry at T1 (*p* = 0.055) and non-stimulated Whole-Saliva Sialometry at T2 (*p* = 0.058). There were no significant correlations in T3–5.

There were no correlations between whole-saliva sialometry with and without stimulation and specific taste sensation during the course of RT (Table 2). 

### EORTC quality of life questionnaires

#### EORTC C30

Comparison between questionnaire results were divided between questions 1–28 and 29–30, due to the different scales used. Between T0 and T1, there was a declining trend (*P* = 0.058) of 1 point for question #29 “*How would you rate your overall health during the past week?*”, but no significant difference with regards to question #30 “*How would you rate your overall QoL during the past week?*”. When both questions were analyzed together, there was a downward trend of 1.05 points (*P* = 0.058). Between T0 and T2, for questions #1–28 assessing QoL, there was a significant decline of 0.26 points (*P* = 0.013) in the average responses.

#### EORTC HN43

Between T0 and T2, there was a significant decline of 0.38 points in the average response in EORTC HN43 (*P* = 0.001).

For question #45 “*Have you had problems with your sense of taste?*” there was a significant difference of 1.38, 1.61, 1.3 and 0.7 points between T0 and T1, T2, T3 and T5, respectively (*P* = 002, 0.004, 0.01 and 0.023 respectively). There was a significant correlation between the decline in objective taste sensation and subjective patient assessment of taste sensation between T0 and T2 (*P* = 0.024).

#### EORTC C30 & EORTC HN43

When both questionnaires were summed, there was a significant decline of 0.32 points in the average response, between T0 and T2 (*P* = 0.001).

### Correlation between sialometry and QOL questionnaires

At T1 - There was a significant negative correlation between question #42 “*Have you had a dry mouth?*” and Whole-Saliva Sialometry with and without citric acid stimulation (*P* = 0.013 and *P* = 0.031, respectively); the higher score for subjective xerostomia, and lower saliva volume in sialometry.

At T2, there was a significant negative correlation between questions #42 and #43 “*Have you had sticky saliva?*” and Whole-Saliva Sialometry with and without citric acid stimulation (*P* = 0.002 and *P* = 0.002; *P* = 0.018 and *P* = 0.005, respectively), with the higher score for subjective “sticky saliva”, and the lower saliva volume in sialometry.

At T4, there was a trend towards correlation between question #42 and stimulated Whole-Saliva Sialometry (*P* = 0.083).

At T5, there was a significant negative correlation between question #42 and sialometry with and without citric acid stimulation (*P* = 0.003 and *P* = 0.044, respectively), and between and question #43 and sialometry without citric acid stimulation (*P* = 0.034).

## Discussion

This is a prospective study of the effect of radiotherapy on taste sensation and its relation to saliva production, unique in its use of validated objective and subjective tests. In the case of H&N cancer, taste sensation impairment can be caused by tumor or surgery-related loss of taste buds, even before starting RT. In fact, taste impairment has been documented at baseline by several studies [10, 11, 30]. For this reason, we chose to compare the change in taste sensation during and after RT to individual patients’ baseline. We found a significant decline in overall taste sensation at mid-point (T1) and end of RT (T2) course, compared with baseline (T0). Moreover, we found statistically significant declines with specific tastes such as sweet and salty, a trend with bitter, and no change with sour. The median decrease in subjective taste sensation as evaluated by QoL questionnaires (question #45 in EORTC HN43) correlated with these results. At later time points, 1- and 3-months post radiotherapy (T3 and T4, respectively), no significant differences were found from baseline, indicating recovery of taste sensation.

Previously published reports on variations in taste acuity have had conflicting results. For example, several studies have found that bitter and salty tastes were affected early and more severely [6, 7, 10, 14]. Another study found that sour taste was significantly impaired after radiation, while bitter, salty, and sweet tastes were not [3]. The reason for these discrepancies is unknown. The use of citric acid in sialometry in our study could potentially mask alterations in sour taste. However, patients had to rinse with distilled water between sialometry and the taste strip test and wait several minutes. Patients also had to rinse after each specific taste strip. As sour was the second taste tested, the patient has already rinsed at least three times before the first exposure (between sialometry and taste strips, after a control strip and a sweet strip). Thus, we do not believe stimulated sialometry affected our results.

The underlying mechanisms for acute and chronic taste loss have been a matter of debate. Damage to the taste buds rather than nerve damage has been suggested by some to be the cause for the rapid recovery of taste sensation [14]. In one study, the numbers of lingual fungiform papillae were assessed in a patient and control groups at the start of the study and after exposure to radiation up to 2 months. The patient group showed a loss of lingual papillae that recovered by 6 months to the same level as the control group [3]. In another study, laser-scanning microscopy was used to compare taste buds and epithelia of fungiform papillae of healthy subjects with those of patients suffering from taste disorders during or after radio-chemotherapy. A significant decrease of taste function was associated with thicker epithelia and smaller areas of the taste pores [31]. Other studies have also reported on the association between the number of taste buds and taste acuity [32]. It has been suggested that long-term taste losses could be related to damage to neural tissues such as the lingual and glossopharyngeal nerves [6]. While we did not find a correlation between taste and mucositis during RT, patients who had prolonged severe mucositis did have a significantly worse overall taste sensation. This finding may be related to an overall slower healing rate of acute toxicity. Interestingly, while the objective tests did not detect a significant difference after recovery from each patient’s baseline, patients continued to subjectively report “*a problem with teste sensation*” (question #45 in EORTC HN43) at all study assessment points, including T5, and although its severity decreased, this finding was statistically significant. The objective tests could not determine whether this finding could be attributed to psychological causes or to actual minor deviations detected.

Radiation dose and distribution, and its effect on taste alteration have also been a matter of research. Some studies found significant associations between taste dysfunction and mean radiation doses to oral cavity and to the anterior tongue [3, 5]. The authors hypothesized that damage to taste receptors and minor salivary glands when mean oral cavity dose rises is responsible for this finding [3, 5]. Another study found that taste loss was independent of radiation dose [1]. We did not find any significant correlation between the max or mean dose to the oral cavity and overall taste sensation. As previous reports have found that even relatively low doses in the region of 30–45 Gy can induce dysgeusia, this may not be surprising [9, 15]. Other studies suggested a correlation between taste loss and the volume of irradiated tongue [2, 31]. We did not find significant correlations between doses to different areas of the tongue and overall or specific tastes. However, a larger cohort would be required in order to determine the effect of RT on different anatomical areas of oral cavity and tongue specifically.

We hypothesized that radiation associated deficits in taste sensation may be related or affected by damage to the salivary glands and associated xerostomia. However, some previous reports have not found such a correlation [3–6]. It is also unclear what is the most significant contributor to long-term xerostomia. One study found no correlation between the volume of irradiated parotids and dysgeusia [2]. As there are several contributors to saliva production, in our study we chose to focus on Whole-Saliva Sialometry rather than doses to specific salivary glands or collection of saliva from specific glands. We found a statistically significant positive correlation between sialometry and baseline overall taste sensation. We also found strong trends towards a correlation between sialometry findings and overall taste sensation, meaning that in cases where saliva secretion was objectively lower with or without stimulation, patients’ taste sensation was rated worse. Potentially, with a larger cohort, these trends could become significant.

Recovery time for taste is also a matter of debate. Our study has found a rapid recovery by 1 month following treatment in overall taste sensation as well as specific taste qualities. Similar findings were found in a study that assessed the sense of smell and all four taste qualities at baseline, during 6 months and 1 year after head and neck RT [14]. Taste thresholds for all qualities were elevated at 1-month during RT, and returned to baseline levels at 6 months. Olfactory function was unaffected, as the olfactory receptors were outside the irradiated field [14]. In a prospective questionnaire-based study that also assessed hypo-ageusia during and after H&N RT or chemoradiotherapy, the maximum values were reached during the seventh week of RT, declined a month later, and approached baseline at 6 months from end of treatment [12]. Another study found that the highest prevalence of taste loss was seen 2 months after RT [7]. Some studies found incomplete recovery of taste after therapy, even at late timepoints [3, 6, 7, 9], and even beyond a year after completion of the RT course [3]. The reason for the early recovery of taste sensation in our study is unclear. One potential explanation is relatively low mean doses to the oral cavity in our cohort. The impact of other factors could also be of importance, and require further investigation. Such factors include patients’ age, diet, treatment planning method, radiation dose and distribution, smoking status etc. Assessing their effect would probably require a larger cohort.

Our study has clear limitations. Its relatively small sample size may have led to sampling bias. Also, while we excluded patients who had undergone total glossectomy, had diseases affecting saliva secretion, had a reported history of abnormal sense of taste or eating disorders, or were previous or current heavy smokers, we did not perform correlations with Body Mass Index (BMI), tumor location and other factors that could affect alterations in taste or saliva production. Our study has some clear strengths. Patients were treated in a single center, followed prospectively and assessed by a limited number of medical staff using validated objective methods.

## Conclusions

Our study found significant reduction in taste sensation that rapidly improved after treatment completion. We found strong trends to a correlation with saliva production that requires further exploration. While our study did not find a correlation between doses to the uninvolved oral cavity and taste dysfunction, results from other studies suggest “as low as reasonably achievable” (ALARA) is a good approach.

## Data Availability

The datasets used and/or analyzed during the current study are available from the corresponding author on reasonable request.

## Abbreviations

H&N: Head and neck
RT: Radiation therapy
QoL: Quality of life
Gy: Gray
CT: Chemotherapy
CTCAE: Common Terminology Criteria for Adverse Events
EORTC: European Organisation for Research and Treatment of Cancer
IMRT: Intensity-modulated radiation therapy
MLC: Multileaf collimator
VMAT: Volumetric modulated arc therapy
ALARA: As low as reasonably achievable
